# Investigating practices and difficulties in communicating with patients about COVID-19 vaccination among healthcare workers in Italy

**DOI:** 10.1038/s41598-025-88581-5

**Published:** 2025-02-20

**Authors:** Giorgia Della Polla, Grazia Miraglia del Giudice, Raffaele Cirillo, Vincenza Sansone, Francesco Napolitano

**Affiliations:** Department of Experimental Medicine, University of Campania “Luigi Vanvitelli”, Via Luciano Armanni 5, 80138 Naples, Italy

**Keywords:** Communication, COVID-19 vaccination, Healthcare workers, Italy, Survey, Disease prevention, Health services

## Abstract

The aims of this cross-sectional study were to understand the healthcare workers’ (HCWs) practices and difficulties in communicating with patients about COVID-19 vaccinations, to investigate the factors associated, and to identify targets to improve the efficacy of the COVID-19 immunization strategy. Questionnaires were administered between November 2021 and March 2022 in three immunization centers in Italy. More than half of HCWs (56.8%) reported to always recommend COVID-19 vaccination to their patients, and the recommendations for other vaccinations were provided by 50.4% of the participants. Physicians/medical residents, males, and those who recommended other vaccinations to their patients were more likely to always recommend COVID-19 vaccination. The participants’ perception of difficulties in communicating with patients about COVID-19 vaccination and the impact of sources of information on patients’ knowledge about vaccination, explored using a ten-point Likert-type scale, resulted in a mean value of 6.3 and 7.9, respectively. A higher level of perception regarding difficulties in communicating with patients was more likely to be found among nurses/midwives and younger HCWs. It is important to reduce HCWs’ perceived gap regarding difficulties in communicating with patients, supporting them through health policy to recommend vaccinations, and engaging them in increasing uptake rates.

## Introduction

It is well known that SARS-CoV-2 infects people of all age groups. However, older individuals and those with comorbidities such as diabetes, chronic respiratory and cardiovascular diseases are at a higher risk of developing severe complications^1^. Moreover, the possible emergence of new variants underlines the need to achieve targeted COVID-19 immunization coverage and to support vaccination campaigns for booster doses^2^. Indeed, even in countries that have attained relatively high coverage, the need for additional doses of new vaccines targeting different variants or further booster doses indicates that vaccination campaigns are far from over^3,4^. In Italy from September 2021, the COVID-19 vaccination booster dose was available and free of charge with priority for groups of population at higher risk such as individuals with chronic medical conditions, those aged 60 and over, patients in long-term healthcare facilities, healthcare workers (HCWs), and pregnant women^5^. Worldwide, given the significant variability in COVID-19 vaccination acceptance rates^6–10^, it is important to underline the crucial role of HCWs in influencing the patients’ decisions^11,12^. Indeed, different studies have shown that the main reasons for the COVID-19 vaccine hesitancy include having insufficient information about the vaccine and concerns about its safety^3^. Moreover, public understanding of the role of COVID-19 vaccination in preventing severe forms of the disease is low in Italy^13^. Therefore, those offering the vaccination are responsible for ensuring that individuals understand its benefits, as well as potential side effects. Physicians, being at the forefront of community healthcare, play a crucial role in advising the public on COVID-19 vaccination and their willingness to recommend the vaccination and their skills in communicating effectively are crucial to the success of immunization programs. Nevertheless, physicians often miss the opportunities to provide effective recommendations^4,14^, and they frequently experience difficulties in communicating the importance of vaccinations during their clinical practice^15^.

Several studies were conducted about HCWs’ attitudes and practices in recommending COVID-19 vaccination, but there is limited literature exploring the physicians’ difficulties in communicating on this topic^16^. Therefore, to address this gap, this cross-sectional study conducted in Italy aims to understand HCWs’ attitudes and practices and possible challenges to communication with patients about COVID-19 vaccination, to investigate the factors associated, and to identify targets to improve the efficacy of the COVID-19 immunization strategy.

## Results

A total of 442 HCWs out of the 710 selected completed the questionnaire for a response rate of 62.2%. Internal consistency reliability assessed using Cronbach’s α was 0.81. The main socio-demographic and professional characteristics, and information regarding self-reported vaccinations coverage are summarized in Table 1. Participants had a mean age of 41.5 years, 266 were female (60.6%), 216 were married or cohabiting (49.8%), and 266 respondents had no children (61.7%). 273 participants were physicians or medical residents (62.8%), and 39.8% worked in an emergency/surgical ward. Regarding self-reported vaccination coverage, the vaccine against hepatitis B was the most received (52.7%), followed by tetanus-diphtheria-pertussis (45.7%), while only 19% of HCWs stated having received the influenza vaccine during the last season. Slightly 9.9% of the sample reported having received all the recommended vaccinations for HCWs. 195 respondents knew the mortality rate of COVID-19 at the time of the survey (49.7%), and only 16.9% correctly indicated the chronic medical conditions associated with a higher risk for severe SARS-CoV-2 infection.

**Table 1:** Main socio-demographic and professional characteristics of the sample, and information regarding vaccinations coverage.

Characteristics	*N*	%
Age, in years	41.5 ± 11.5 (23–70)*	
Gender		
Male	173	39.4
Female	266	60.6
Marital status		
Married/cohabiting	216	49.8
Unmarried/separated/divorced/widowed	218	50.2
Profession		
Nurses/midwives	162	37.2
Physicians/medical residents	273	62.8
Working area		
Emergency/surgical	176	39.8
Other	266	60.2
Length of working activity, in months	87.3 ± 118.4 (1–516)*	
Self-reported vaccination coverage		
Hepatitis B	233	52.7
Tetanus-diphtheria-pertussis	202	45.7
Mumps-measles- rubella	161	36.4
Varicella	161	36.4
Seasonal influenza	84	19

Number for each item may not add up to the total number of the study population due to missing value.

*Mean ± standard deviation (range).

HCWs showed positive attitudes toward COVID-19 vaccination. The great majority agreed or strongly agreed that COVID-19 vaccination was useful for patients with chronic medical conditions (94.8%) and for HCWs (93.4%), and that it was effective (92.3%) and safe (85.5%). Moreover, 91.6% and 88.6% of the participants agreed or strongly agreed that COVID-19 vaccination should be mandatory for patients and HCWs, respectively.

A total of 239 respondents always recommended COVID-19 vaccination to their patients (56.8%), whereas 214 participants (50.4%) recommended other vaccinations (hepatitis B, tetanus-diphtheria-pertussis, mumps-measles-rubella, varicella, and seasonal influenza). Multivariate logistic and linear regression analyses identified several predictors significantly associated with the different outcomes of interest (Table 2). The results of Model 1 showed that physicians/medical residents (OR = 2.08; 95% CI = 1.01–4.32), males (OR = 0.51; 95% CI = 0.26–0.98), and those who recommended other vaccinations to their patients (OR = 32.43; 95% CI = 16.21–64.88) were more likely to always recommend the COVID-19 vaccination (Model 1 in Table 2).

**Table 2:** Results of the multivariate logistic and linear regression analysis describing the determinants of the different outcomes of interest.

Variable	OR	SE	95% CI	*p*
**Model 1.** HCWs who always recommend the COVID-19 vaccination to their patients Log likelihood = −131.71, χ^2^ = 188.02 (11 df), *p* < 0.0001				
Recommending other vaccinations to their patients	32.43	11.47	16.21–64.88	< 0.001
Males	0.51	0.17	0.26–0.98	0.045
Physicians /medical residents	2.08	0.77	1.01–4.32	0.049
No need of additional information regarding COVID-19 vaccination	0.63	0.21	0.34–1.18	0.152
Believing that COVID-19 vaccination is safe	2.43	1.73	0.61–9.79	0.213
Not having received all recommended vaccinations	0.54	0.27	0.21–1.45	0.22
No knowledge of which chronic medical conditions increase the risk of getting SARS-CoV-2 infection	0.63	0.26	0.28–1.43	0.27
Believing that COVID-19 vaccination should be mandatory for HCWs	2.53	2.39	0.39–16.13	0.325
Not perceiving difficulties in communicating with their patients regarding COVID-19 vaccination	0.92	0.08	0.78–1.09	0.341
Believing that COVID-19 vaccination is useful for patients with chronic medical conditions	0.35	0.39	0.04–3.24	0.353
Younger	0.99	0.01	0.96–1.02	0.399

	*ß* **coeff.**	SE	*t*	*p*
**Model 2.** Perceiving difficulties in communicating with their patients regarding COVID-19 vaccination F(5, 206) = 4.46, *p* = 0.0007, R^2^ = 9.77%, adjusted R^2^ = 7.58%				
Younger	− 0.05	0.01	− 4.32	< 0.001
Nurses/midwives	− 0.87	0.36	− 2.37	0.019
Having acquired information from scientific journals regarding COVID-19 vaccination	0.59	0.32	1.87	0.063
Working in emergency/surgical area	0.44	0.29	1.47	0.142
Need of additional information regarding COVID-19 vaccination	0.28	0.28	0.99	0.324

The participants’ perception of difficulties in communicating with patients about COVID-19 vaccination and the impact of sources of information on patients’ knowledge about vaccination, explored using a ten-point Likert-type scale, resulted in a mean value of 6.3 and 7.9, respectively. The main reasons described for difficulties in communicating were media disinformation (55%), patients’ low level of education (33.6%), and that patients did not need additional information regarding COVID-19 vaccination (20.7%). The results of the multivariate linear regression model showed that a higher level of perception regarding difficulties in communicating with their patients was more likely to be found among nurses/midwives and younger HCWs (Model 2 in Table 2).

Lastly, 412 HCWs had received information about the COVID-19 vaccination (98.3%), and the most utilized sources were scientific journals (58.3%), followed by the Internet (46.3%), colleagues (43.6%), mass media (34%), and scientific meetings (28.3%). Half of the respondents (50.1%) expressed the need of additional information about the COVID-19 vaccination.

## Discussion

To our knowledge, this study is the first aimed to analyze the determinants of difficulties in communicating identified by HCWs during COVID-19 vaccination campaign in Italy. The findings revealed important information regarding four key points such as HCWs’ recommendations of the COVID-19 vaccine to the patients, HCWs’ knowledge of COVID-19, reported difficulties in communicating with patients, and the sources used to acquire information.

First, more than half of the HCWs recommended COVID-19 vaccination to their patients, whereas recommending other vaccinations was less frequently reported. This result is similar to previous investigations conducted in Europe and Asia^17–19^ but lower than studies conducted during the first two doses campaigns in Italy^14^ and in other countries^20,21^. This could be explained considering that the booster dose was administered for the first time to HCWs just before the interview, so this probably influenced their behavior in recommending the vaccination. Moreover, the findings of previous studies conducted in Western countries during the first two doses campaigns showed that HCWs were more likely to recommend a vaccine they got in the past^18–20^. Finally, the multivariate logistic regression analysis showed that physicians/medical residents and males were more likely to recommend the COVID-19 vaccination to their patients and it is interesting to note that these variables were previously found as predictors of HCWs’ willingness to receive the vaccination^22^. HCWs’ changes in attitudes and behaviors regarding vaccinations should be investigated with repeat surveys during different phases of an infection spread, and this may be a focus for future investigations.

Second, regarding HCWs’ knowledge of COVID-19, less than half were aware of the COVID-19 mortality rate at the time of the survey, and only 16.9% correctly indicated all chronic medical conditions associated with higher risk for severe COVID-19. Improving HCWs’ knowledge of diseases and related vaccinations is essential, in order to positively influence vaccination uptake and willingness, as described in different target groups of population^23–26^. In particular, it is widely established that greater knowledge about COVID-19 vaccination leads to less vaccine hesitancy among HCWs, parents, and the general population, influencing their willingness to receive the vaccine^17,27,28^.

Third, HCWs reported some difficulties in communicating with the patients about the COVID-19 vaccine due to media disinformation (55%), patients’ low level of education (33.6%), and reluctance to get information (20.7%). The issue of difficulties in communicating between HCWs and patients has been discussed about COVID-19 vaccination and previous investigations are consistent with the results of this investigation^29–32^. This study reinforced the importance of the physicians’ role in communication with patients to improve positive behaviors and attitudes such as vaccination uptake and willingness, as described in the past literature^33,34^. Moreover, the results of the multivariate regression analysis showed that the good practice of recommending COVID-19 vaccine to patients is more frequent among physicians or medical residents, as already reported in previous studies^18,19,35^. A higher level of perception regarding difficulties in communicating with patients was reported by nurses/midwives, who may experience major difficulties in recommending vaccination, since this is mainly part of the physicians’ training and practices^36,37^. Indeed, a recent survey showed that physicians more than other HCWs are in favor of reinforcing the mandatory COVID-19 vaccination for themselves and their patients^38^. These findings confirm that physicians represent a well-informed group of HCWs and play a key role in improving vaccination coverage. It is important to underline the necessity of supporting interventions to improve training programs on vaccination for all HCWs in order to better spread the knowledgeable information about recommended vaccinations for the patients at higher risk. Another interesting finding is that younger HCWs were more likely to perceive difficulties in communicating with patients and this could be explained assuming a lower level of knowledge about recommended vaccinations^32^, less experience, and fewer years of clinical practice^39,40^.

Fourth, regarding the sources of information, the most reported were scientific journals followed by the Internet. Scientific journals represent the highest point of reliable information consulted by HCWs to improve their knowledge and practice^41,42^. However, although most respondents indicated sources of information that are recognized as credible and of quality such as scientific journals and meetings, a relevant number of HCWs indicated potentially unreliable source of health data, such as internet and mass media that can spread inconsistent information. Acquiring information in this way can be worrisome, as it is known that the unofficial information about vaccinations available on the Web may be false or not evidence-based^43–45^. Fake news and other misleading information may increase the HCWs’ concerns about vaccines’ efficacy and safety, negatively affect their appropriate practices on the immunization recommendations and, consequentially, patients’ decisions to follow preventive measures, representing a potential health harm. Therefore, the exclusive consultation on the Internet of official sources of information from public health authorities universally recognized as reliable and digital health literacy developed among HCWs could reduce this critical issue^46^.

The results from the present study should also be considered with some potential methodological limitations. First, the cross-sectional study design cannot establish causal relationships between the independent variables and the different outcomes of interest. Second, the data were collected in immunization centers in a single geographic area, and therefore, the findings cannot be generalized to all Italian HCWs. Third, a self-reporting questionnaire may have introduced social desirability bias and the surveyed HCWs may report more positive attitudes and practices that lead to an overestimation of their perceived difficulties in communicating about COVID-19 vaccine and related recommendations. However, an anonymous questionnaire has been used to reduce this bias. Fourth, the investigation was conducted during the first pandemic waves and, therefore, the study findings may not apply in COVID-19 endemic seasons. Despite these limitations, this study was the first to assess the HCWs’ perceived difficulties in communicating with patients regarding COVID-19 in Italy, and it provides important information for health policymakers to improve the efficacy of immunization programs.

## Conclusions

In conclusion, the results of this study reveal important points concerning difficulties in communicating about vaccination. First of all, it is clear that the knowledge of HCWs need to be implemented to spread accurate information to patients relating vaccination. Secondly, it is important to be aware of the need to bridge the perceived gap regarding difficulties in communicating with patients, especially among the youngest HCWs who should be supported through health policy to recommend vaccinations and effectively engaged in increasing the vaccination coverage. Targeted training programs, improved access to reliable resources, and communication skill development activities of HCWs should be prioritized to address these challenges in order to improve the efficacy of the immunization strategies.

## Materials and methods

### Study design, participants, sampling procedures, and data collection

The current study was part of a larger project conducted in the Campania Region, in the Southern of Italy, during the COVID-19 pandemic^41,42,47^. The study was carried out between November 2021 and March 2022 and a two-stage cluster sampling method was used to recruit the participants. In the first stage, three immunization centers in the Campania region, including autonomous vaccination facilities developed during the pandemic and hospital-based facilities, were randomly selected. In the second stage, a random sample of HCWs (including physicians, medical residents, nurses, and midwives), attending the immunization centers from Monday to Friday, was selected. The sample size was calculated considering that 50% of respondents recommended the COVID-19 vaccine to their patients, using a confidence level of 95%, and a margin of error of 5%, resulting in a sample size of 384.

Before starting data collection, the Directors of the selected immunization centers received a letter requesting collaboration in the survey. Once the approval to conduct the study was obtained, HCWs attending the immunization center were approached by the investigators who explained the purposes and importance of the study, that the participation was non-mandatory, the information was collected anonymously and confidential, and no data could assist in identifying the respondents. The study did not require any time limits, compensation, or incentives for participants. Informed consent was obtained from all participants before the start of the survey.

An anonymous self-administered questionnaire that was built ad hoc for the study, consisting of five sections, was used to collect information. Internal consistency was calculated with Cronbach’s α. The first section aimed to investigate the socio-demographic and professional characteristics of the participants such as age, gender, educational level, professional role, working area, and to gather information regarding self-reported vaccinations coverage against hepatitis B, tetanus-diphtheria-pertussis, mumps-measles-rubella, varicella, and seasonal influenza. The second section explored the knowledge about the COVID-19 infection, such as mortality rate and risk factors. The third section focused on the HCWs’ attitudes and general concerns regarding COVID-19 and related vaccination through six statements, measured with a five-point Likert-type scale ranging from 1 (strongly disagree) to 5 (strongly agree), including the perceived usefulness of vaccination for HCWs and patients, the belief that the vaccination should be mandatory, and concern about its safety and efficacy. In the same section, a ten-point Likert-type scale, ranging from 1 (not at all) to 10 (at all), explored the HCWs’ opinion about the perceived difficulties in communicating with patients, the impact of information sources on patients’ awareness about COVID-19 and the related vaccination. The fourth section collected information regarding the HCWs’ practices about COVID-19 and other recommended vaccinations (hepatitis B, tetanus-diphtheria-pertussis, mumps-measles-rubella, varicella, and seasonal influenza), measured with a five-point Likert-type scale from “never” to “always”. In the last section, participants were asked to indicate their sources of information regarding COVID-19 vaccination and their willingness in receiving further information.

To assess the questionnaire’s comprehension, expected completion time, and face validity, a pilot study was conducted among 20 participants, which resulted in some changes to enhance the instrument. The overall analyses did not include the answers from these respondents.

The study was conducted in accordance with relevant guidelines and regulations and approved by the Ethics Committee of the Teaching Hospital of the University of Campania “Luigi Vanvitelli”.

### Statistical analysis

The participants’ characteristics and responses to different items were investigated using descriptive statistical analysis. The continuous variables were measured using means and standard deviations, while the categorical variables were measured using frequencies. Afterwards, the chi-square test or Student *t*-test were applied in univariate analysis to evaluate the relationship between categorical and continuous variables and their outcomes of interest, respectively. The following independent variables, considered potential determinants, were included in the stepwise multivariate logistic and linear regression models performed to investigate which of the different characteristics were associated with the following outcomes of interest: HCWs who always recommend the COVID-19 vaccination to their patients (Model 1) (no = 0; yes = 1); perceiving difficulties in communicating with their patients regarding COVID-19 vaccination (Model 2) (continuous). To improve models’ specificity, the significant level of the p-value for the inclusion and elimination of the variables in the models was set at 0.05 and 0.4, respectively. The following variables were included in all models: gender (male = 0; female = 1), age in years (continuous), profession (nurses/midwives = 0; physicians/medical residents = 1), working area (other = 0; emergency/surgical = 1), having received all recommended vaccinations (no = 0; yes = 1), having acquired information from scientific journals regarding COVID-19 vaccination (no = 0; yes = 1), and need of additional information regarding COVID-19 vaccination (no = 0; yes = 1). The variables knowledge of which chronic medical conditions increase the risk of getting SARS-CoV-2 infection (no = 0; yes = 1), believing that COVID-19 vaccination is effective (strongly disagree/disagree/uncertain = 0; agree/strongly agree = 1), believing that COVID-19 vaccination is safe (strongly disagree/disagree/uncertain = 0; agree/strongly agree = 1), believing that COVID-19 vaccination is useful (strongly disagree/disagree/uncertain = 0; agree/strongly agree = 1), believing that COVID-19 vaccination should be mandatory for HCWs (strongly disagree/disagree/uncertain = 0; agree/strongly agree = 1), believing that COVID-19 vaccination should be mandatory for patients with chronic medical conditions (strongly disagree/disagree/uncertain = 0; agree/strongly agree = 1), believing that COVID-19 vaccination is useful for patients with chronic medical conditions (strongly disagree/disagree/uncertain = 0; agree/strongly agree = 1), recommending other vaccinations to their patients (no = 0; yes = 1), and perceiving difficulties in communicating with their patients regarding COVID-19 vaccination (continuous) were included in Model (1). Moreover, the variable HCWs who always recommend the COVID-19 vaccination to their patients were included in Model (2). For logistic regression analysis, odds ratios (OR) and their 95% confidence intervals (CI) were provided for interpretation, while for linear regression analysis, ß coefficients and standard errors were given. Statistical significance was defined as *p*-values less than 0.05 in all analyses. STATA 18 software was used to analyze the collected data.

## Data Availability

The raw data supporting the conclusions of this article will be made available by the corresponding author, without undue reservation.
